# Mouse Bio-behavioral Phenotyping Using a Digital Homecage Framework for Long-timescale, High-resolution, and Multi-factor Data Collection and Analytics

**DOI:** 10.21203/rs.3.rs-9152533/v1

**Published:** 2026-04-17

**Authors:** Nicolette Ognjanovski, Anjesh Ghimire, Pho J Hale, David S Kim, Ivo H Cerda, Ethan Goldiez, Simeone Marino, Antwan Green, Paul J. Fitzgerald, Priya Vijayakumar, Deniz Kirca, Matthew Tong, Noah Muscat, Kennedy Knopf, Mitchell Cook, Mingyi Tang, Yang Chen, Lezio Soares Bueno Junior, Ridge Weston, Tangyu Liu, Jeremiah P. Hartner, Ivo D. Dinov, Brendon O. Watson

**Affiliations:** Michigan Medicine; Michigan Medicine; University of Michigan–Ann Arbor; Michigan Medicine; Harvard University; Michigan Medicine; Michigan Medicine; Michigan Medicine; Michigan Medicine; Michigan Medicine; Michigan Medicine; Michigan Medicine; Michigan Medicine; Michigan Medicine; Michigan Medicine; University of Michigan–Ann Arbor; University of Michigan–Ann Arbor; Michigan Medicine; Michigan Medicine; Michigan Medicine; Michigan Medicine; University of Michigan–Ann Arbor; Michigan Medicine

## Abstract

Long-term monitoring of behavioral and physiological processes is essential for elucidating complex brain-based phenomena and disorders that develop over extended periods, such as chronic stress, circadian disruption, and metabolic syndromes. Though digital phenotyping is well-established in humans via smart devices, comparable solutions for rodent models remain limited. To address this gap, we present the Digital Homecage (DHC) system, an open-source platform that enables uninterrupted, long-timescale recording of over 20 behavioral metrics in single-housed mice. The DHC integrates video capture, operant task modules, and wheel-running data to achieve sub-second resolution in monitoring behaviors such as actigraphy, sleep, grooming, and food choice over periods spanning weeks. Initial data validate the system’s capability to reveal circadian patterns across multiple spontaneous behaviors, consistently reflecting nocturnal activity. Designed for seamless integration with brain recording technologies, the DHC offers unique opportunities for longitudinal analyses of chronic conditions and neuropsychiatric syndromes. Its accessibility and modular design promote community-driven innovation, establishing the DHC as a transformative tool for advancing the study of complex traits in rodent models of brain function and behavior.

## Introduction

Rodent neuroscience tools hold promise for studying brain-based phenomena and disorders, but many such phenomena develop over long periods or require long-duration monitoring to capture [1–4]. Such slow-developing conditions include chronic stress, circadian disorders, aging, metabolic disorders, and others [5–8]. Unfortunately, the slow development of those conditions means their behavioral phenotypes are not fully characterized by standard short-term behavioral tests commonly used over recent decades. Furthermore, when those standard tests are administered repeatedly, they often show changing behavioral results due to stress, acclimatization, or other factors. On the other hand, non-invasive measures of spontaneous behavior in the homecage can be performed indefinitely for the lifetime of the animal and can reveal long timescale dynamics without interference.

In an approach called “digital phenotyping,” human researchers use digital devices such as smartphones or wrist-based trackers to gather behavior and physiologic data, often over weeks or months. Using this longitudinal approach, scientists have discovered novel associations between behaviors and mental health states [9–14]. In animal laboratories, scientists are positioned to gather the same type of longitudinal daily activity data about rodents, since animals live in facilities over weeks and months; however, behavioral testing typically occurs for only a few minutes or hours per week. Thus, rodent neuroscientists stand to gain vast amounts of information by recording rodent homecage behavior 24 hours per day [15].

To automatically capture such 24-hour rodent data we set out to create “rodent digital phenotyping”. While some homecage-based systems already exist, they often are not optimized for assessment of broad ranges of behavior or are not easily modified to modern recording/manipulation tools, such as electrophysiological recordings or optogenetic systems [16–21]. Such systems have often also been wholly commercial in nature [21, 22]; whereas computer control systems, 3D printing techniques, and open science approaches now allow new lanes of innovation to individual labs such that they are no longer confined to commercial products. Additionally, the “big data” gathered from long-term recordings of rodents are now more easily stored and processed, and using, machine learning techniques can be brought in to complement more traditional analytics.

The “Digital Homecage” (DHC) framework introduced here uses a combination of videographic, operant, and running wheel data to capture 20 + behavioral metrics from a single-housed mouse at sub-second resolution, without interruption, over weeks. Broadly, this system can measure sleep, actigraphy, voluntary exercise, grooming, and exploration, as well as various consummatory behaviors. These are coupled with physiological assessment throughout the course of the experiment, providing the potential for detailed longitudinal analyses. This automated system will allow new observations that define correlates of chronic conditions and is made freely available via online repository.

## Results

### Digital behavior phenotyping system overview

We aimed to develop a platform capable of automatically recording a wide range of spontaneous murine behaviors in a standard laboratory homecage setting for weeks or months at a time. We prioritized affordability and accessibility including use of a standard rodent strain, relatively low cost ($2800) for most components. Video processing software for behavioral classification is a major bifurcation point and we have designed the system to enable either option: users can either adopt an open source system which requires manual training (LabGym, DeepEthogram or others - free) [23,24] or use an expensive commercial system (HomeCageScan (HCS), CleverSys, see Table S1), as we did in this publication. The simple, modifiable design, standard parts, and compatibility was designed with anticipated future, long-term brain recordings or manipulations. Our main objective was to provide an easily customizable interface at a fraction of the cost of a commercial design, with which other investigators can design their own experiments. For this reason, the DHC was designed for two main modes: behavior-only (which we present here) and behavior + electrophysiology, for which many of our design decisions were made to include in the future.

To promote generalizability across labs, we designed our system to record from individual C57BL/6J mice (Jackson Labs, JAX:000664), the most commonly used mouse strain, using a standard manufacturer homecage (Allentown) with simple modifications. This cage is surrounded by a system of operant feeders and water dispensers, camera, and a running wheel inside the homecage (Fig. 1). For this publication, we designed and built 16 fully operational DHC units (Fig. 1a), capable of chronically recording daily spontaneous activity, and report behavior-only mode.

The external box enclosing the cage and equipment is made of polyvinyl chloride (PVC) foam boards and is light-tight when closed, with a hinged top door to allow easy access for daily welfare checks and maintenance (Fig. 1a). The DHC utilizes Standard commercial Allentown mouse cages modified to include automated food and water dispensers, and a running wheel (Fig. 1b) This modified cage is placed within the PVC panels and surrounded by monitoring and feeding devices. The biological and environmental variables controlled within this DHC experiment are shown in Fig. 1c and data stream types in Fig 1d. To create a standard circadian light cycle, we diffused LED strip through a sheet of translucent white plexiglass such that a light meter placed at the center of the mouse cage measured ~40 lux (with added bedding and a ventilated cage lid) on a 12 hour:12-hour light:dark (LD) cycle.

To enable 24-hour video recording of behavior during both light and dark periods, an infrared LED strip, which illuminated outside the mouse’s visually perceivable range, was attached to the walls of the PVC panels behind a flat white acrylic diffuser. A camera with an infrared filter was placed on the opposite side of the cage, resulting back-lit side-view recording of behavior. The camera records continuously at 30 frames/second and generates video files that are later processed with software for video-based actigraphy analysis. Additionally, an automated, high-throughput behavior classification pipeline is used to identify dozens of different behaviors (e.g., grooming, rearing, sleep) at sub-second resolution. This can be performed either with HomeCageScan (CleverSys), as shown here, or may also be possible with the open source LabGym platform after manual training of this AI-based system. We have initiated such training and while it is work-intensive, there is no software cost. Non-video measures are at collected (sampled? Idk how the new software works) at 16Hz and include nose pokes and beambreaks for two operant feeders and two operant water dispensers, as well as the rotary position of a running wheel to gather exercise-related data.

All measures for a given animal/DHC including camera frames are synchronized to the clock of a single computer, such that all data streams for a given animal are recorded relative to the same clock. This computer time is later converted to real-world POSIX timestamps for ease of processing. Video and food/water/wheel recordings are restarted together each 4 hours to reduce any drift or mitigate any file corruption. Data collected by the DHCs are supplemented by experimenter-executed daily software and animal welfare checks including weight, food and water refills, and intermittent fur coat score and cage changes [25].

We chose to focus on maximal behavioral detail from each individual animal. Therefore, we chose to single-house the mice; otherwise we would only have cohort-level accuracy. This single housing will also enable future phenotyping of mice with cranial implants for electrophysiology, optogenetics, or otherwise. Given their prior correlations with stress, measures of sucrose water preference (versus regular water) and fatty food preference (versus regular chow) were integrated into the DHC [26–28]. To measure this, sucrose and regular water were placed into the two respective water dispensers and regular versus fatty food into the two food pellet dispensers. To control for and measure any location preference for the food or water dispensers, positions of sucrose versus regular water were swapped every 3 days and fatty versus regular food swapped every 7 days (Fig. 1e, top). Given the added stressor of single housing, we closely monitored stress signatures via daily physical checks [29]. A common measure of mouse stress is fur coat score (Fig. 1e, bottom), which was recorded manually every 3 days, coincident with water change days, so as not to add additional interactions with the mouse. As both overgrooming [1] or under-grooming [7] create visible markers of animal distress, we also compared fur coat score during the first 2 weeks of acclimation relative to the following 2 weeks of baseline. We observed no statistical changes in fur coat score at any of the 6 positions assessed across the 28 days (p=0.4184, ANOVA: main effect of time, n=1422 across all body segments).

Table 1 shows features of our DHC relative to other digital phenotyping systems. The DHC system has a variety of strengths relative to these and advances the field. Using our DHCs, we have recorded activity of 16 mice at a time, continuously for 8 weeks. We have not found specific temporal limitations on duration of recording but here we will present initial month-long data.

### Experimental recordings

Sixteen wildtype male C57BL/6J mice were recorded for 4 weeks (28 days). This provided adequate time for animals to normalize behavior to the novel environment. In the rest of this publication, these 28 days are broken down into 2 weeks “Acclimation” followed by 2 weeks “Baseline” (as annotated in Fig. 2 and beyond). Data are binned and analyzed relative to the lights-on zeitgeber (ZT0). Our completed DHC records three main data streams: operant food and water choice, running wheel use, and 24/7 video. While other automated animal monitoring systems have presented similar analysis for activity [15,17,20,30], the DHC records these three components concurrently at high resolution (Fig. 2).

For food and water monitoring, we allowed mice the choice between 1% sucrose water versus regular water and fatty food (15%) versus regular food pellets (Fig. 2ai). There are four beambreaks, one for each of two food choices and two water choices, and each animal can see both left and right choices at all times (Fig. 2aii, right). Mice had continual access to all food (blue, shown as a total of fatty and regular) and water (red, shown as a total of sucrose and regular) across the duration of the experimental timeline (Fig. 2aiii). We found that some mice repeatedly poke for food or water without consuming what is dispensed and even creating cage flooding. Therefore, we set a 3-second minimum time between dispenses (this timeout period is user-configurable). When measuring around-the-clock data, we find that dispense events are approximately 2/3 as frequent as nose pokes.

To track running behavior, a common commercial rodent running wheel (Bioserv, Mouse Igloo) is modified with a 3d printed clear dome to prevent mice from crawling under the wheel, thereby protecting a 3-pin micro connector carrying an analog wheel position signal to a LabJack T7 device. The wheel’s angular position is encoded via a rotary encoder, which reports a range of 0 to 5V at a sampling frequency of 16Hz (Fig. 2bi) with each voltage change denoting angular change of the wheel such as from running (Fig. 2bii). Running events or ‘bouts’ were defined as a sequence of distance measurements separated by gaps ≤ 2 seconds that were measured in each hour of daily recording. Per-minute cumulative distance across the experiment was calculated for each DHC, and abnormal periods (absence of running or sustained continuous) were compared to available video data, and animal activity that did not match reported distance values were replaced with ‘NaN’ and excluded from analysis. We find that it takes mice 7±1.25 days to begin continuous, daily use of the running wheel as seen by cumulative distance travelled (Fig. 2biii).

We recorded video data at 30 FPS using a Basler camera (Fig. 2ci), although the frame rate can be increased or decreased by the user. In Fig. 2cii we show both raw IR videos and extracted animal outlines for running and interaction with the dispenser representing eating or drinking. Fig. 2ciii displays an example of the top 15 behaviors characterized by HCS as well as LabJack wheel data for four days (representative animal). These behaviors, for example ‘turn,’ are summations of other more nuanced behaviors that fall under that classification, such as ‘turn right’ or ‘turn left.’ We observe consistent time-of-day (light vs. dark) effects on behaviors that are coincident with published reports of nocturnal behavior [31,32].

### Multi-timescale analysis

Outputs of all DHC digital data streams can be analyzed at time scales ranging from seconds up to weeks. We assessed the three main data streams from the DHC (as shown in Fig. 2), food and water consumption, running, and video analysis in bins of weeks, days, and hours with emphasis on 24-hour circadian timepoints across the day-night cycle (Fig. 3).

For food and water consumption, we analyzed beambreaks (to represent the behavioral action of the mouse as opposed to relying on proper dispense functioning) for 1% sucrose (pink) and regular (blue) water as well as regular (green) and fatty (orange) food consumption (Fig. 3a). First, we examined weekly averages. In Fig. 3ai, vertical dotted line shows the difference between the 2 weeks of acclimation and 2 weeks of baseline for all plots. Mice on average nose poked for sucrose water 3x as much as they sought out other food or drink options (13.93±1.14 pokes per hour for sucrose water compared to regular water [2.78±0.31], regular food [4.08±0.45], and fatty food [3.91±0.53], p<0.001, Browne-Forsythe ANOVA, Dunnett’s T3 multiple comparison for sucrose vs all others). Fatty food was not requested or dispensed significantly more than regular food (p>0.999, Browne-Forsythe ANOVA, Dunnett’s T3 multiple comparison). We next plotted daily data, allowing us to account for monitoring, food/water swaps, and cage changes as well as to improve temporal resolution (Figs. 3aii, also S4). Milestones of this particular experimental paradigm are shown with dashed vertical lines in each graph: water swap days are shown by blue lines and food swap days by red lines. Animals show overall preference for sucrose water over regular water (Fig. 3aii) but seem to exhibit a place-preference in the positioning of their food, with clear swapping of fatty vs regular food eating coincident with food position swap days (complete statistics shown in Table S2). This will be further analyzed in the next section. We lastly quantified hourly feeding and drinking around the 24-hour cycle averaged over days, to show circadian patterns. Mice, being nocturnal, eat and drink almost exclusively at night, with most dispenses occurring during the dark phase across all food and drink options (Fig. 3aiii, regular water: ZT16.97 ±0.34, sucrose water: ZT18.29 ±0.16, regular food: ZT17.64 ±0.15, fatty food: ZT17.74 ±0.17).

We then characterized weekly, daily, and circadian wheel running data (Fig. 3bi-iii). Weekly averages (Fig. 3bi) show a majority of animals (n=12) level off of running behavior after the 2-week acclimation period running ~7 km/day (Figs. 3bii, S5). On average the mice run 1.805 km/day during acclimation versus 7.39 km/day average during baseline days (p<0.001, paired t-test for first 14 days compared to last 14 days, full statistics see Table S2), which is consistent with free-running behaviors in populations of wild mice [33–35]. This suggests that under laboratory constraints, with any possible stress due to single-housing, mice do not behave naturalistically until 14+ days of consistent exposure to the running wheel. We next analyzed data within repeated 24-hour daily cycles, in hourly bins, and averaged across animals to create circadian plots of daily running rhythms (Fig. 3biii). The average distance run within each hour shows that this cohort of animals ran mostly at the beginning of the dark period (Fig. 3biii, green line) which is the expected active phase for the nocturnal mouse. We quantified the mean vector for this running activity for all recorded animals (Fig. 3biii, black arrow) and find maximum activity around ZT15.67 ±0.08. This may coincide with bursts of activity at the start of the active phase after 12 hours of increased sleep.

For video analysis, following HCS software-based scoring of multiple behaviors from video, we summated across the two weeks of baseline recording and generated a pie-chart displaying percentage of total video frames spent in each behavior across all (n=16) animals for all days for which we ran HCS (Fig. 3ci). After grouping the 44+ behavioral classifications provided by HCS as described in methods, we found that the top 15 behaviors account for 92.76 ± 0.43% of all time recorded. We quantified these top 15 behaviors in daily bins, with bar graph portions indicating total time spent in each behavior across the day (Fig. 3cii). Sleep accounted for 43.19% of average daily behavior, the largest behavior across all days of HCS analysis. Over these 14 days, the percentage of time varies slightly on a weekly cycle and sometimes around water swap day (Figs. 3cii and S6). These results reflect an expected amount of inter-individual variability.

We then quantified circadian phase of behaviors across all animals using a mean resultant vector of the hourly occupancy for each behavior (Fig. 3ciii, colors same as in 3ci). While most behaviors occurred during the dark (active) phase, the “sleep” and “twitch” behaviors (Fig. 3ciii, orange and periwinkle arrows, respectively) occurred during the light (inactive) phase (ZT4.50 ±0.22 and ZT4.99 ±0.24, respectively for sleep and twitch). This is of note as twitches are often associated with REM sleep or sleep more generally [36–39]. This preference of sleep-based behaviors in the inactive phase then serves as initial validation of the video scoring methodology. Furthermore, the phase (ZT18.12 ±0.22) of the “eat” behavior via video scoring was similar to the phase of food consumption using our operant feeder data (as shown in Fig. 3aiii, ZT17.69 hours average eating time across both food types). HCS calculated average drinking occurs at 17.98 ±0.22, which is again similar to the water beambreak timing of about ZT17.5 hours. These time-of-day preferences validate the accuracy of the commercial automated behavior scoring algorithm relative to hardware tracking of these actions.

### Preferences in animal feeding behaviors

Mice show preference for sucrose water over regular water (Fig. 3a) despite water location swaps, but weekly food location swaps sometimes correlated with swaps in fatty versus regular food consumption (Fig. 3aii). Therefore we analyzed more by summing beambreaks for both water and food types each day across the full population of animals (Figs. 4a-b, respectively). We observed swapping peaks for each position, in alignment with mice following the sucrose water dispenser position (Fig. 4a) as well as a preference for food at position 2 (Fig. 4b). Across the 4 weeks of acclimation and baseline, the total amount of water, independent of position remains stable (5.28mL/day, average 2.634 ±0.205 mL for position 1 and 2.648 ±0.226 mL for position 2, p= 0.9508, paired t-test for entirety of recording). This data is not shown for food, as the DHC cannot confirm number of pellets dispensed and ultimately eaten, only the request and successful dispense of pellets. Mice have been seen on video requesting multiple pellets and not consuming them but rather letting them pile up in front of the dispenser.

During our 4-week recording time, mice lost weight across the first 3 days, possibly due to familiarizing themselves with the feeding setup and running wheel. Generally, weight rebounded starting on day 4 and stabilized into the baseline period (Fig. 4c, 29.95g ±0.137g, p= 0.0624, Welch’s ANOVA test for average weight over time).

To account for swapping the position of water every 3 days, we quantified food and water nose pokes in position 1 (closer to the power source) versus position 2 (closer to the diffuser panel and the IR light) regardless of which type of water was at each position. We also quantified by water/food type. First, we observed an overall preference for sucrose (p< 0.0001, paired t-test) across all animals, regardless of dispenser position (Fig. 4d, left, blue=regular water and pink=sucrose, as in previous figures). This was shown by findings of significantly non-zero but opposing values for position 2 minus position 1 regardless of sucrose position – the opposing values showing that sucrose was more preferred than position (Fig. 4d, right). Using related logic, sucrose minus regular water was positive regardless of sucrose position (Fig. 4f, right), again indicating that sucrose preference overrides place preference for water.

There was no preference for food type (Fig. 4e, left, p=0.6618, paired t-test). In the test of position 2 minus position 1 for food data, we found a consistently positive value regardless of which position had fatty food (Fig. 4e, right), indicating that position preference overrides type preference for food. Relatedly, a subtraction of fatty minus regular food did depend on position of fatty food (Fig. 4g, right, p< 0.0001), meaning position was more important than food type. These behaviors could be studied more deeply in future DHC experiments.

### Web-based data processing module

We built a DHC data framework as an integrated development environment (IDE) based on the statistical language R. This allows us to utilize sophisticated backend data analytics R libraries, statistical learning, and AI tools, as well as RShiny frontend user and machine interfaces. Both video- and feeder/water/wheel-based data are initially saved in un-binned format with fully precise, sub-second timestamping. R code then bins data into days, hours, and minutes for presentation and analysis online (Fig. 5). On the RShiny server, users are able to view data via a web browser interface for quality control or basic assessment.

The server has two major modules: One focused on time bin-based displays (Fig. 5a) and another on statistics and population activity (Fig. 5b). The RShiny app supports real-time interactive online visualization supporting quick exploration and identifications of potential failures in either the hardware or software stream. Data can be viewed at the individual animal level. This interface also allows export of both graphics and spreadsheet-format versions of data, binned at minute, hour, and day for further analysis using other software programs.

## Discussion

The DHC system facilitates high-throughput, sub-second recording of multiple spontaneous behaviors across several animals simultaneously, enabling precise digital phenotyping with both high temporal and behavioral resolution. Its customizable interface empowers users to collect and analyze longitudinal data spanning multi-week interventions, daily paradigms, circadian rhythms, and sub-second events. We specifically focus on spontaneous behavior given the frequency and importance of spontaneous behavioral changes in neuropsychiatric syndromes in humans [40, 41]. Thus, this high-throughput and high-resolution approach can establish integrative biomarkers for disease models or other long-developing temporal processes.

We have been able to consistently use the DHC over multiple weeks in 16 mice simultaneously. We validate our system by observing significant circadian modulation of many daily behaviors including actigraphy, sleep, eating, drinking, wheel running and foraging. Importantly, we observed that wheel running requires approximately 14 days to reach a steady state, as well as feeding and drinking behaviors taking several days to normalize after experimenter interventions like water swap (place preference differences) and cage changes (video differences). This could be a matter of learning the wheel initially and then building the interest and habit, or perhaps there is a delay for mice to become comfortable with the DHC thus delaying naturalistic running tendencies. This demonstrates that while shorter behavioral paradigms may not capture steady state behaviors, the DHC can easily do so - with important implications for future work, since many measures may be best performed when animals are in a steady state.

We alternated food and water placements to assess the consistency of preference for regular vs fatty food and regular water vs sucrose water. We found that in general there is a preference for sucrose water over regular water, but no preference for fatty vs regular food. The majority of animals did prefer a specific food dispenser, possibly driven by the initial locations of each option. Preference for position may result from unobservable effects, but position 2, for which mice show preference, is located closer to the IR light diffuser panel in our design. Although IR light has been shown to not effect overall feeding or drinking[42], it has been shown that mouse retinal cone cells can be activated by IR light[43]. Perhaps mice detect a luminance difference. This preference could also reflect mouse handedness, or preference to turn clockwise vs counterclockwise, for which individual mice have shown biases [44, 45]. On the other hand, sucrose water was consistently preferred despite location swaps. These data suggest high inter-individual variability across such a long recording time that may be explained by other factors such as running or behavioral changes.

The DHC monitors a family of metabolic factors including weight, dietary choices, and exercise, including the relative balance between each. We saw consistent weight over the last two weeks of the recordings, implying self-regulation of caloric status despite changing content including increased calories through sucrose water, ability to choose fatty versus regular food, and the new opportunity to exercise with a running wheel.

Additionally, time-of-day quantifications are efficient with this system with many behaviors showing modulation by time of day. For example, wheel running and sucrose water consumption occurred most in the early dark hours. On the other hand, the behaviors sleep and twitch happen mostly in the light hours in this nocturnal animal. The distinction between sleep and idle behavior is an age-old problem and the reason we built the DHC for the future incorporation of cranial EEG is so that we or others can more precisely measure sleep around the clock and across weeks. Grooming was among the least circadian modulated behaviors, as it occurred relatively constantly in every hourly time bin (average 6.99 ± 0.134 minutes each hour) with no clear preference for day or night as observed in most other behaviors.

There are numerous avenues of analysis to follow up on using such large data sets from the DHC which has a number of advantages compared to other user-made homecages (see Table 1). First, it can quantify baseline behavioral attributes of individual animals, therefore capturing inter-individual variation and possibly providing predictors of subsequent responses to interventions. Second, it can lead to data-driven multi-dimensional markers of response to stress, drug treatments, or other interventions. This stands in particular contrast to traditional experimenter-initiated tests that do not capture natural behavior and are often conducted at arbitrary times. Third, it can also lead to markers that are based on temporal evolution of multiple elements. For example, after chronic psychosocial stress sleep changes could precede alterations in eating or grooming.

Many neuropsychiatric syndromes, and indeed most behavior in general, are based on self-motivated, spontaneous drives which are often well-monitored at home. A homecage-based approach can thus behaviorally complement experimenter-defined assays; as a result, some researchers have studied more naturalistic circadian time-scale changes in the homecage [15, 17, 46]. Indeed, many biological studies rarely acknowledge baseline fluctuations, more so a comparison of changes from baseline. Not only does the DHC measure these spontaneous behaviors, but with future development of the intracranial recording/manipulation aspects of our system it can also probe the mechanisms of those phenotypes using powerful rodent neuroscientific tools such as electrophysiology, optogenetics, or others.

Overall, the DHC may be applied more broadly after the initial development and characterization of the baseline recordings of spontaneous activity presented here. Behavioral tendencies, sex differences, or finding stereotyped behavioral sequences (e.g. nesting before sleep or drinking before eating) will all provide new fundamental data for mouse neuroscience [47]. Beyond baseline homecage behavior in wildtype mice, behavioral questions can be examined using the DHC including drug and stress response, optogenetic or chemogenetic interventions in specific brain circuits, genetic mutant behavior changes, and others. Additionally, the DHC as presented is currently being scaled up to accommodate housing for rats (*Rattus norvegicus*) and other rodents.

## Methods

All animal husbandry and surgical/experimental procedures were approved by the University of Michigan Institutional Animal Care and Use Committee (PRO00011423-BOW). Male C57BL/6J mice (n = 16; IMSR_JAX:000664, Jackson) were individually housed in standard caging modified for the DHC (see details below). All methods were performed in accordance with the relevant guidelines and regulations and are compliant with the Essential 10 from The ARRIVE guidelines 2.0 as detailed within the individual subsections below. Mice were between 3–6 months old for the duration of the experiment. Environmental lights were maintained on a 12:12 light:dark cycle (lights on at Zeitgeber Time [ZT]0) with food and water available ad libitum. Prior to the start of each experiment, bedding and running wheels were added to each mouse cage in each DHC and the brightness of the visible LED was calibrated. Every 7 days, cages were replaced with clean cages with fresh bedding. Maintenance and care, including institutional permission and oversight information for experimental animal use were overseen by University Laboratory for Animal Medicine (ULAM) at the University of Michigan with approval from IACUC.

## Box Design

### Exterior Encasement:

A 68.6 cm × 55.9 cm × 43.2 cm box made with white inch Foam PVC (American Plastic Solutions) encloses the DHC system. The enclosure has a 25.4 cm × 25.4 cm (10” by 10”), light-tight louver vent in one side wall that maintains temperature, air composition, and humidity to an acceptable level, offsetting the heat produced by the various electronic components and light sources that the system deploys.

### Mouse Cage:

A standard commercial animal cage (Allentown, #PC75JCL) with standard bedding (Nestlets, LabSupply) provides the housing for our chronic measurements. Two 3.8 cm × 5.1 cm figure-eight shaped patches matching the shape of our two dispensing units were drilled out of the Arduino-facing side of the mouse cage (one of the smaller walls of the rectangular cage). A small 1.27 cm hole is taken from the bottom of the cage to introduce the 3-pin micro mating connector of the Running Wheel System. Mice are allowed to eat, drink, run, engage in other behaviors, and sleep *ad libitum*. Prior to the start of each experiment, bedding and running wheels were added to each mouse cage in each DHC and the brightness of the visible LED was calibrated.

#### Lighting and Schedule

We used a 12:12 light:dark cycle for these experiments, although the schedule can be changed via LabJack code. All visible LEDs were on daily at 05:00 UTC for 12 hours, and IR LEDs for camera illumination are on 24-hours daily, diffused through a #7328 Translucent White Acrylic Sheet. Visible light is emitted from 6 LEDs that output 6000 Kelvin light (to simulate bright daylight) at approximately 40 lux encased within a rectangular prism of white translucent acrylic that measures 0.635 cm × 34.3 cm × 61 cm). 40 lux brightness is set via Light Dimmer ZDM-01 from JKL Components and is measured at the center of each mouse cage with the mouse cage lid attached.

#### LabJack

There is one computer for each DHC, connected to a camera and a LabJack T7 digitizer (with a CB15 Terminal). The LabJack timestamps events and continuously stores them in two .csv files on the DHC-specific computer to which it is connected by USB. Food and water beambreak and dispense data go to one file, while running wheel data go to a different .csv. For food and water, LabJack records as binary (digital) the TTL voltage outputs from the Arduino: half of channels recording beambreak times and half recording dispense commands to water and food motors/solenoids. LabJack records as analog (16-bit) the 0–5 V from the running wheel rotary encoder. All channels specified in Fig.S1b. For each event, line is appended to the end of a .csv which includes a POSIX time entry in the first column. Additionally, we employ the LabJack T7 5V DC output to precisely control the light cycle of the boxes as described in the Visible Light Relay Assembly subsection. The computer for each DHC also writes .mp4 video files from the camera for each DHC and stores them to a local RAID. All files are automatically backed up daily to distant cold storage.

#### Electrical System Design

The Circuit Panel Assembly takes in 12V of power from the AC/DC converter (Mean Well USA Inc, EDR-75–12) and distributes it to key components of the DHC (as shown in Fig. S1) including the Light Relay Station, the Arduino, and the Motor Shield. The Circuit Panel Assembly consists of a 1) Power Distribution Board Assembly, 2) 12V Arduino Barrel Jack Wire Assembly, 3) Buck Converter Power Assembly, 4) 12V Visible Light Relay Power Wire Assembly.

##### 1) Power Distribution Board:

the right half of a red Breadboard Mini contains the system’s 12 VDC power rail, which is supplied directly by the high-capacity power supply which gets power from the wall. The Arduino, the infrared backlight, and the visible LEDs all operate directly from this 12V source. On the other hand, the Motor Shield Assembly also draws from this supply after it is stepped down to 9V-DC via a buck converter (LM2596, Valefod). The left half of the breadboard contains the independent Water Solenoid Valve Driving Circuit, which allows the Arduino Motor Shield to control the opening and closing of solenoid valves for water dispensing.

##### 2) 12V Arduino Barrel Jack Wire Assembly.

This assembly consists of a 53 cm-long cable connected to the Power Distribution Board on one end and a male barrel jack connector on the other end. It provides 12V DC power to an Arduino Mega on the Arduino-Motor Shield Construct.

##### 3) Buck Converter Power Assembly.

The buck converter steps down the 12V input from the Power Distribution Board to provide 9V of power to the Motor Shield. It consists of a 10 cm-long 2-conductor cable connecting the Power Distribution Board’s 12V and GND rails to the input terminal of a buck converter that steps down the voltage output to 9V. A 72 cm-log wire then connects the buck converter’s output to the Motor Shield Assembly, supplying 9V of power.

##### 4) Visible Light Relay Assembly.

Made of a 51 cm-long 2-conductor cable running between the Power Distribution Board’s 12V and GND rails to a 5V relay board’s NO and COM pins set to low trigger (COM-15093). The removal of a 5V digital input coming from the LabJack T7 during the transition from the 12 night-hours to the 12 day-hours completes the circuit and turns on a constant lux LED strip to simulate daylight conditions. The NO pin of the relay board and the positive lead of the 9 cm (3.5 inch) long LED strip are connected via a female-male barrel jack junction. The LabJack T7 provides the power relay board with 5V of input during the 12 night-hours and removes it during the 12 day-hours. In the absence of that 5V input, the power relay board supplies 12V of power to the visible light LED strip.

#### Arduino-Motor Shield Construct

This consists of an Adafruit Motor Shield Assembly (1438 v.2) mounted on an Arduino Mega 2560 (Product number A000067). DC power is split as described above, with 12V for the Arduino and 9V for the stepper motor output of the mounted Motor Shield. The shield also uses 5V from the Arduino to power its logic on-board.

Arduino Mega 2560 links beambreak inputs at the nose poke ports to the water or food dispensing outputs to be mediated by the Motor Shield (opening valves or powering motor rotations). The Arduino code can be calibrated to control the water dispense drops of water via modification of the time the solenoid valve remains open. Also a “timeout” function in the code ensures dispenses cannot happen with an interval of less than 3 seconds regardless of nose pokes in the 3 second refractory period. Regardless of the timeout configuration, Arduino records all beambreak events and relays the information to the LabJack T7 via an IDC parallel cable. All dispense events are also recorded to LabJack.

In the Motor Shield Assembly, the white, red, and green wires of 6 female 3-pin connectors (CAB-14575) are soldered onto their corresponding inputs in the digital I/O rail, power rail, and ground rail of an Adafruit Motor Shield V2, respectively. Four (4) of these connectors interface with the 4 different Beambreak Assemblies (sucrose and regular water, fatty and regular food). Soldering the connectors to the Motor Shield Assembly instead of the Arduino allows our system to remain modular, and enables the Arduino to easily be reprogrammed, assessed, and swapped out if needed. The Motor Shield receives a 5V digital input in the event of a beambreak at the dispensing ports, and according to I/O relationships encoded by the Arduino, it either uses 9V to energize one of the coils in the appropriate stepper motor or sends a 5V digital logic input to the Nchannel enhancing type MOSFET of the solenoid driving circuit. This input allows current flow through the appropriate solenoid valve for a user-defined period, resulting in water dispensing. An IDC parallel cable transfers digital information encoding all beambreak and dispensing events from the Arduino to the LabJack T7, which also directly receives analog data encoding running wheel revolutions.

#### Food Dispensing and Water Dispensing

##### Dispensing Structure:

The 3D-printed structure was made using Prusa MK4 with PLA/PETG resin (Overture) or Formlabs2 3D Printer, rigid resin (FLRGWH01) and is composed of one main body holding two stepper motors to rotate each of two food pellet hopper wheels and two food holders above the hoppers, two chutes for hopped food to descend to the mouse, and a mouse interface printout. The mouse interface includes four nose poke beambreaks, two receptacle catches for each of the food chutes, and water dispenser needles for water dispense. This design was based off of an open-source design from https://openephys.atlassian.net/wiki/spaces/OEW/pages/79069188/Food+Pellet+Dispenser. This was followed by our own modifications after converting the .stl files into editable SolidWorks files. To enable this, Allentown mouse cages were modified with two holes to accommodate two food dispensing modules and two water dispensing modules as mentioned above.

The food dispensing works via a chute that delivers food from an elevated receptacle when the mouse pokes its nose in the beambreak of the port. A servo motor turns to drop the food down the chute to a food presentation portion of the mouse port – a few millimeters below the food beambreak zone. The upper dispenser under the receptacle has slits to prevent debris accumulation (Fig. 2ai). Two water ports are similar, dispensing either regular or 1% sucrose water (Fig. 2aii, left). These work via a nose poke beambreak leading to the opening of a solenoid to let water flow down a tube from a suspended syringe.

##### Beambreak Assembly:

Our system employs four Beambreak Assemblies, one at each dispensing port, consisting of an optical switch photointerruptor relying on infrared light (Mouser Electronics 474-BOB-09322), a male 3-pin connector that interfaces with the female connectors of the Motor Shield Assembly, and a 220Ω current-limiter resistor to protect the infrared LED. The Motor Shield receives a 5V digital input in the event of a beambreak, and according to I/O relationships encoded by the Arduino, it either uses 9V to activate one of the coils in the appropriate stepper motor or sends a 5V digital logic input to the N-channel enhancing type MOSFET of the solenoid driving circuit.

##### Food:

Regular (4 mm diameter, 3.8% fat) pellets and 15% purified medium-fat pellets (Bio-Serv Dustless Precision Pellets F0165 and F0021, respectively) are provided *ad libitum* for the mouse via the two separate food dispensing units located inside the mouse cage. When a nose poke is detected by the Beambreak Assembly at one of the food-dispensing ports and the digital signal is processed as outlined in the Arduino-Motor Shield Construct, the Motor Shield ends up supplying 9V, 500 mA to a corresponding bipolar, 2-coil stepper motor (Pololu-1208, SY35ST28-0504A) located in the 3D printed Dispensing Structure. The stepper motor has a 1.8° step angle, offers a holding torque of 0.09 Nm, and is connected to the Motor Shield via four wires, two for each motor coil. The stepper motor shaft is lodged into the pellet distributor disk and is lined with eight, 0.635 cm (1/4 inch)-diameter, evenly spaced holes that fit a single pellet at a time. To prevent clogging of the system with pellet debris, the receptacle base is lined with rectangular slits, and the direction of motor rotation is reversed once every 5 steps via Arduino code.

##### Water:

Filtered water and 1% sucrose water are provided *ad libitum* for the mouse via the two separate water dispensing ports inside the cage. Two 10 mL syringe shafts (Bio-Rad: M10LLASSM), one filled with 1% sucrose water and the other with distilled regular water, are connected to the input port of the corresponding solenoid valve via a 76 cm-long, 1.6-mm ID Tygon water tube (Bio-Rad: 7318215) fitted with a female lure lock adaptor on the syringe shaft end. The open end of a similar 10-inch-long tube connects to the output port of the solenoid valve. The other end arrives at the water dispensing port and is fitted with a male lure lock adaptor and a 20-G × 1.5-inch needle (Medline SYR100207) with the bevel removed and tip dulled to prevent any injuries to the mouse. The Motor Shield supplies 5V of constant power to the Water Solenoid Valve Driving Circuit. The circuit also includes a Zener diode (1N4757A) for overvoltage protection, a rectifier diode (14005-T) for forward biasing, and a 10 kΩ (CF14JT10K0CT-ND) pulldown resistor.

#### Running Wheel System

The system uses a commercially available mouse running wheel (Bio-Serv Fast Trac and Mouse Igloo K3250) made of autoclavable amber polycarbonate transparent to infrared light, preventing it from blocking our infrared camera. The interface between the igloo and the trac was modified to accommodate a stainless steel, 1.07 cm (0.42 inch)-diameter shaft coupler (ServoCity: 625152) fitted with a wooden 4.7 mm (3/16-inch) diameter × 3.56 cm long wooden Dowel rod that lodges into the Fast Trac on one end and a MA3 Miniature Absolute Magnetic Shaft Encoder (US Digital MA3-A10-125-B) on the other. This analog output voltage is recorded by our LabJack by means of a 4-conductor shielded round cable with a 3-pin micro mating connector on one end (CA-MIC3-SH-NC unterminated with shielded cable, US Digital).

Discrete events are refined from raw voltage outputs from each animal’s DHC. The wheel running system records a voltage between 0–5V which corresponds to the wheel’s absolute angular position anytime the voltage changes. And once the wheel is in full motion with the animal running on it, 180 degrees between LabJack entries (16Hz/60ms). To determine when running events occur, we used MATLAB to analyze the output files from each DHC for significant voltage changes and related these changes to assumed running events, assigning them a distance based on the circumference of the wheel and how many revolutions occurred in one instance of running behavior.

Relative voltage was then multiplied by 0.07690168, the distance in meters traveled around the circumference of the wheel associated with Δ1V recorded by the wheel running system. For each output file (1 per day per DHC), distances were accumulated over a 1 second window (or until the end of the data was reached) and assigned a timestamp. Running events were defined as a sequence of distance measurements separated by gaps ≤ 2 seconds. For each running event, a start time, end time, distance, and speed were all recorded for each animal. From this list of events, intervals were defined as the difference between successive start times and averaged for each animal under different conditions by filtering across timestamps: average across the entire experiment, average during lights on/off, and average per day using the same methodology as described for the beambreak and dispense intervals.

#### Infrared Video Recording System

Our system makes use of a camera (Basler, acA1300–200 um) with infrared-pass filter (FRMS208 Nir Bandpass filter, Machine Vision Store) for which we record at 30 fps at 640 × 512 resolution. The camera connects to a desktop computer via USB and produces an .mp4 video file every 4 hours. For video imaging, animal and homecage are illuminated by an infrared LED backlight that remains perpetually lit behind a translucent plexiglass panel that diffuses the light, allowing for illumination at the infrared camera. The wavelength of the infrared light falls below the detection threshold for mice, allowing for continuous recording, even in dark hours. Visible light is modulated daily, while this infrared light remains on constantly. BandiCam software is used to record frames reliably and also to start and stop acquisition every 4 hours. Our system can record videos at various levels depending on the needs of the experiment. While Bandicam allows for screen recording, it also allows for recording video from an external camera. For experiments where video-based behaviors are desired, but high temporal resolution is not necessary, Bandicam serves as a cheap, robust, crash-resistant option. Timestamped video files are then analyzed with both HomeCageScan (CleverSys) and MATLAB code to extract video-based actigraphy.

In experiments where temporal resolution and frame timestamping are essential, for example– future electrophysiological recordings, GPIO pins on the recording cameras can be used to output a pulse for each frame. This gpio pin inputs into a Digital Input pin on a recording interface (here we used the Intan RHD USB recording system, but others like the Open Ephys acquisition board can also be used). For these experiments, we chose to use Streampix8, as each copy of Streampix supports multiple cameras (we used 4x cameras, each recording from a separate DHC). Physical wiring and software setup for this are being uploaded to the Camera page of the DHC wiki. Saved to local RAID and then transferred every 24 hours to University cold storage, with a network connection not being required for initial saving to RAID.

#### Software Ecosystem

Arduino Integrated Development Environment (IDE): contains code to control the Arduino-Motor Shield Construct. Its functions determine input-output relationships in the Motor Shield underlying food and water dispensing. This allows testing and manipulating the operation of the stepper motors and solenoids, diagnosing their failure causes, and troubleshooting potential issues with functions that can, among other things, unjam clogged dispensing mechanisms.

phoBehavioralBoxLabjackController: This custom software written in C + + collects running wheel revolutions, beambreak and dispense event data, and controls the toggle of the warm yellow LED strip via the LabJack T7. The program produces output in the form of two .csv files, one for digital data and the other for analog data.

LabJack custom software: Writes a new line to the digital (food/water nose poke/dispense) or analog .csv file whenever a voltage change is detected. In each line, the full state of all digital or analog pins is written using Booleans (digital) or 0–5 voltage (analog) alongside POSIX timestamps. The LabJack T7 uses a rate of 50 kHz.

#### Digital Clock Synchronization:

Each of our embedded devices operates on its own internal clock. Desktop computers come with an accurate real time clock built-in, which is frequently synchronized with the NIST atomic clock. This allows POSIX timestamping of data from each stream to enable time-of-day analyses.

#### Quantitative Analysis and Statistics

All analysis (food/water/running wheel/video) was cleaned and organized as described below and data were then converted to zeitgeber time (ZT) with hour ZT0 at lights on and hour ZT12 at lights off. The default value for each analysis was hours; however, for continuous actions such as wheel running, minute data were also extracted for duration measurements. All analyses were automated therefore blinding for any user bias.

All data cleaning and pre-processing is done via outlined protocols on the Github and at https://drive.google.com/file/d/1yC9Oqn0sPjQ8LcdTdSg0OnXRPiFu6BPj/view?usp=sharing., with details for each data stream provided below.

##### Food and Water:

Using Matlab (Mathworks, 2024b) we binned (by hour) digital data for beambreak and dispenses from the RShiny server as separate CSVs for each animal across the experimental period. First, new rows were added for hours which had no recorded beambreaks/dispenses and a count of 0 assigned. Across all DHCs, hours with more than 500 beambreaks were noted and investigated for dispenser clogging or leaking. If the video for that hour showed dispenser malfunctions or animal activity that did not match beambreak/dispense levels (mouse was sleeping or did not interact with the feeder), both the beambreak and dispense value for that hour were replaced with ‘Not a number’ (NaN) and excluded from all analyses. For analysis by week across animals, for each animal, hourly beambreaks were summed across each week of the experiment for each DHC. For analysis by day across animals, hourly beambreaks and dispenses were summed across each day for each animal then averaged across all animals for each day. For analysis by hour of animals, average was calculated across all available hourly beambreaks and dispenses for all animals for each hour of the day. All data were graphed using GraphPad Prism (GraphPad v.10).

##### Place Preference:

Beambreaks for both food and water types were summed for each day of the experiment across all animals. Values for the daily beambreak sum at each food and water position were parsed from the daily beambreak type values, assigning each daily sum for a type of food or water to the proper position as marked by the researchers in their daily box check. Next, daily differences were calculated across position and type sums, always calculated as (position 2 - position 1) or (fatty/sucrose – regular). For food and water type data, differences were placed in one of two position groups depending on the position of sucrose water or fatty food for that day. For position data, differences were placed in groups depending on what type of food or water occupied position 1 for that day.

##### Running Wheel:

We downloaded minute- and hourly-binned analog data for distance run from the RShiny server as separate CSVs for each animal across the experimental period. Per-minute cumulative distance across the experiment was calculated for each DHC by replacing the value of each cell in the csv with the sum of itself and all values before it. These data were then plotted for each DHC and investigated for non-physiological activity (large spikes or sustained activity during the light period). Abnormal periods were compared to available video data, and animal activity that did not match reported distance values were replaced with ‘NaN’ and excluded from analysis in hour chunks from both binned files for that DHC.

Cleaned data were then converted to ZT in both hour and minute bin files. Per-minute cumulative distance across the experiment was recalculated for each DHC using the same methods as previously described. Similarly to the digital data, new rows were added in the hour binned file for hours which had no recorded wheel running, and a distance of 0 was assigned to that hour. Linear regression was calculated from every hourly binned data point available for the acclimation and baseline periods across each DHC. For analysis done by day across animals, hourly wheel run distances were summed across each day of the experiment for each DHC. For analysis of time per hour spent running, the minute bin entries were counted across each hour of the experiment for each DHC.

To generate vectors, time in hours was first converted to radians to map the 24-hour cycle onto a circle. The vector’s components were calculated by summing the weighted horizontal (x) and vertical (y) contributions of distance or time averages across all hours, using cosine and sine functions, respectively. The mean direction of distance or time was then derived from these components using the arctangent function and converted back to hours. The vector’s magnitude, reflecting the concentration of distance or time around the average hour, was added using vector addition to yield a mean resultant vector, yielding a vector with mean direction and magnitude. This process was performed on each DHC for hourly distance, average distance, and time values across all animals.

#### Video and Behavior–HomeCageScan:

HCS was used for behavioral video analysis from CleverSys Inc (Reston, VA). Videos from the same mouse consecutive in time (usually a week of videos between cage changes) are nominated for batch HCS processing as a “session”-- or indices of the locations of a desired .mp4 video file. Text log files of processing steps for the selected video are saved to assist in later data processing. HCS session initiation requires the outlining of a region of interest to indicate the “cage,” or space wherein the mice will behave, and a static background image to compare movement to. Feeding and drinking zones are also indicated manually as polygons. As video is processed, the software will index for the requested video, apply the virtual cage, and automatically regenerate background images if the program loses track of the animal’s location. Video is processed at maximum speed, 3000 frames per second. Behavioral data are recorded in duration of frames and saved to a .csv file, which is then processed to extract the periods of bouts of certain behaviors. Custom Python software takes in both the finished .csv files, and the text log file containing the video indices and inserts the frame-based POSIX time of each behavior on the .csv.

To create the 14 primary categories analyzed, behaviors in the scored .csvs were combined by similar behavior type (walk left/right/slowly, eat zone 1/2, drink zone 1/2, etc.) across all animals. As described in the text 15 major behaviors characterized by HCS and wheel data for four days are grouped from 44 behaviors. For example ‘turn,’ are summations of other more nuanced behaviors that fall under that classification, such as ‘turn right’ or ‘turn left.’ Fifteen behaviors comprised approximately 93% of animal activity were retained, and the remaining behaviors were grouped in with the existing ‘Other’ category defined by HCS.

Time spent in each behavior per hour was calculated across all available days of data by first iterating over each annotated file, accumulating total durations (converting frames to second) per behavior per hour of the day. The total duration of each behavior per hour was then divided by the number of times that hour occurred in the dataset, returning the time spent in each behavior per hour. Vectors for hourly behavioral activity were calculated using the same methodology as described for the running wheel.

The example 24-hour and two 12-hour behavior breakdown bar charts were calculated by summing the frames spent in each behavior, then converted to seconds based on a consistent frame rate of 30 fps. Total seconds spent in each behavior was divided by 86400 seconds (one day) to determine the percentage of time spent in that behavior.

## Figures and Tables

**Table 1.** Comparison of the DHC to other commercial phenotyping cages. Across 9 different parameters of recording, 11 automated monitoring systems were scored. N/A – May technically be possible, but the possibility lies in the hands of the engineer who designed these, likely with some fairly major changes to the system. Not applicable - impossible and irrelevant to system (FED3 is a feeder). No - Literally impossible due to a technical perspective (usually due to the system requiring group housing)

		Food Tracking	Water Tracking	Video Recording	Running Wheel	Preference Tracking	Time Precision	Typical Experiment Time	Data Time Units	Tether Capabilities
Digital Home Cage	Ognjanovski et al. 2026	yes	yes	yes	yes	yes	sub-milisecond	3+ Months	Minutes, Hours, Days, Weeks	yes
HABITS	Yu et al. 2025	yes	yes	no	no	yes	milisecond	3+ Months	Miliseconds, Seconds, Hours, Days	N/A
ToneBox	Francis et al. 2019	no	yes	no	no	no	33 milisecond	~2 Weeks	Seconds, Hours	N/A
RodentWatch	Hsieh et al. 2024	no	**yes** *	yes	no	yes	~500–1000 ms	>1 Week	Seconds, Hours	N/A
IntelliCage	Lipp et al. 2024	no	yes	no	no	yes	N/A	2–3 Weeks	Seconds, Days	N/A
Automated home cage system	Goulding et al. 2008	**yes** *	yes	no	no	yes	milisecond	1–2 Weeks	Seconds, Minutes, Hours	N/A
PhenoCube	Balci et al. 2013	no	yes	yes	no	yes	33 milisecond	~1 Week	Hours, Days, Weeks	**no** ****
FED3 system	Matikainen-Ankney et al. 2021	yes	no	no	no	**yes** **	milisecond	variable	Minutes, Hours, Days	Not applicable
Automated home-cage system	Jhuang et al. 2010	**yes** *	**yes** *	required	yes	no	frame-based	24 Hours	Minutes, Hours	N/A
DeepEthogram	Bohnslav et al. 2021	**yes** ***	**yes** ***	required	**yes** ***	**yes** ***	frame-based	variable	N/A	Not applicable
PyMouseTracks system	Fong et al. 2023	no	yes	required	no	no	frame-based	variable	Seconds, Days	**no** ****
Smart-Kage	Ho et al. 2023	no	yes*	yes	yes	yes	0.5 second	3+ Months	Minutes, Days	N/A

* Individual dispenses are not detected; measured in time spend at source

** Only place preference is trackable; no type preference

*** Possible with proper training

**** Limited due to group housing

## Data Availability

All descriptions and part numbers provided below are also available via online resource at https://github.com/BrendonWatsonLab/Digital-Homecage/wiki and are continuously updated.

## References

[1] Albrecht A. Neurobiological consequences of juvenile stress: A GABAergic perspective on risk and resilience. Neuroscience and Biobehavioral Reviews 74, 21–43 (2017). 28088535 10.1016/j.neubiorev.2017.01.005

[2] Baldini T. Clinical features and pathophysiology of disorders of arousal in adults: A window into the sleeping brain. Frontiers in Neurology 10, 1–9 (2019). 31164861 10.3389/fneur.2019.00526 PMC6534078

[3] Bonapersona V. The mouse brain after foot shock in four dimensions: Temporal dynamics at a single-cell resolution. Proceedings of the National Academy of Sciences of the United States of America 119, 1–9 (2022). 10.1073/pnas.2114002119 PMC8872757 35181604

[4] Ma P. Fast and slow: recording neuromodulator dynamics across both transient and chronic time scales. Science Advances 10, 1–17 (2024). 10.1126/sciadv.adi0643 PMC10881037 38381826

[5] Logan R. W. Chronic Stress Induces Brain Region-Specific Alterations of Molecular Rhythms that Correlate with Depression-like Behavior in Mice. Biological Psychiatry 78, 249–258 (2015). 25771506 10.1016/j.biopsych.2015.01.011 PMC4509914

[6] Koch C. E., Leinweber B., Drengberg B. C., Blaum C. & Oster H. Interaction between circadian rhythms and stress. Neurobiology of Stress 6, 57–67 (2017). 28229109 10.1016/j.ynstr.2016.09.001 PMC5314421

[7] Acosta-Rodríguez V. A., Rijo-Ferreira F., Green C. B. & Takahashi J. S. Importance of circadian timing for aging and longevity. Nature Communications 12, (2021). 10.1038/s41467-021-22922-6 PMC8129076 34001884

[8] Tran I. & Kathrin A. Long-term effects of chronic stress models in adult mice. Journal of Neural Transmission https://doi.org/10.1007/s00702-023-02598-6 (2023) doi:10.1007/s00702-023-02598-6. PMC10460743 36786896

[9] Jain S. H., Powers B. W., Hawkins J. B. & Brownstein J. S. The digital phenotype. Nature Biotechnology 33, 462–463 (2015). 10.1038/nbt.3223 25965751

[10] Onnela J. P. & Rauch S. L. Harnessing Smartphone-Based Digital Phenotyping to Enhance Behavioral and Mental Health. Neuropsychopharmacology 41, 1691–1696 (2016). 26818126 10.1038/npp.2016.7 PMC4869063

[11] DeBoever C. Assessing Digital Phenotyping to Enhance Genetic Studies of Human Diseases. American Journal of Human Genetics 106, 611–622 (2020). 32275883 10.1016/j.ajhg.2020.03.007 PMC7212271

[12] Montag C., Sindermann C. & Baumeister H. Digital phenotyping in psychological and medical sciences: a reflection about necessary prerequisites to reduce harm and increase benefits. Current Opinion in Psychology 36, 19–24 (2020). 32361334 10.1016/j.copsyc.2020.03.013

[13] Bauza M., Krstulovic M. & Krupic J. Unsupervised, frequent and remote: A novel platform for personalised digital phenotyping of long-term memory in humans. PLoS ONE 18, 1–16 (2023). 10.1371/journal.pone.0284220 PMC10132599 37099501

[14] Coghlan S. & D’Alfonso S. Digital Phenotyping: an Epistemic and Methodological Analysis. Philosophy and Technology 34, 1905–1928 (2021). 34786325 10.1007/s13347-021-00492-1 PMC8581123

[15] Pernold K., Rullman E. & Ulfhake B. Major oscillations in spontaneous home-cage activity in C57BL/6 mice housed under constant conditions. Scientific Reports 11, 1–14 (2021). 33654141 10.1038/s41598-021-84141-9 PMC7925671

[16] Richardson C. A. The power of automated behavioural homecage technologies in characterizing disease progression in laboratory mice: A review. Applied Animal Behaviour Science 163, 19–27 (2015).

[17] Jhuang H. Automated home-cage behavioural phenotyping of mice. Nature Communications 68, 1–10 (2010). 10.1038/ncomms1064 20842193

[18] Fong T., Hu H., Gupta P., Jury B. & Murphy T. H. PyMouseTracks: Flexible Computer Vision and RFID-Based System for Multiple Mouse Tracking and Behavioral Assessment. eNeuro 10, ENEURO.0127–22.2023 (2023). 10.1523/ENEURO.0127-22.2023 PMC10198609 37185293

[19] Goulding E. H. A robust automated system elucidates mouse home cage behavioral structure. Proc. Natl. Acad. Sci. U.S.A. 105, 20575–20582 (2008). 19106295 10.1073/pnas.0809053106 PMC2634928

[20] Matikainen-Ankney B. A. An open-source device for measuring food intake and operant behavior in rodent home-cages. eLife 10, 1–18 (2021). 10.7554/eLife.66173 PMC8075584 33779547

[21] Lipp H.-P. IntelliCage: the development and perspectives of a mouse- and user-friendly automated behavioral test system. Front. Behav. Neurosci. 17, 1–34 (2024). 10.3389/fnbeh.2023.1270538 PMC10793385 38235003

[22] Francis N. A., Bohlke K. & Kanold P. O. Automated Behavioral Experiments in Mice Reveal Periodic Cycles of Task Engagement within Circadian Rhythms. eNeuro 6, 1–10 (2019). 10.1523/ENEURO.0121-19.2019 PMC6775758 31488550

[23] Bohnslav J. P. DeepEthogram, a machine learning pipeline for supervised behavior classification from raw pixels. eLife 10, e63377 (2021). 34473051 10.7554/eLife.63377 PMC8455138

[24] Hu Y. LabGym: Quantification of user-defined animal behaviors using learning-based holistic assessment. Cell Reports Methods 3, 1–16 (2023). 10.1016/j.crmeth.2023.100415 PMC10088092 37056376

[25] Nollet M. Models of Depression: Unpredictable Chronic Mild Stress in Mice. Current Protocols 1, (2021). 10.1002/cpz1.208 34406704

[26] Teegarden S. L. & Bale T. L. Decreases in Dietary Preference Produce Increased Emotionality and Risk for Dietary Relapse. Biological Psychiatry 61, 1021–1029 (2007). 17207778 10.1016/j.biopsych.2006.09.032

[27] Williams E. S. Androgen-Dependent Excitability of Mouse Ventral Hippocampal Afferents to Nucleus Accumbens Underlies Sex-Specific Susceptibility to Stress. Biological Psychiatry 87, 492–501 (2020). 31601425 10.1016/j.biopsych.2019.08.006 PMC7035179

[28] Sequeira-Cordero A., Salas-Bastos A., Fornaguera J. & Brenes J. C. Behavioural characterisation of chronic unpredictable stress based on ethologically relevant paradigms in rats. Scientific Reports 9, 1–21 (2019). 31758000 10.1038/s41598-019-53624-1 PMC6874551

[29] Shin H. S., Choi S. M., Lee S. H., Moon H. J. & Jung E.-M. A Novel Early Life Stress Model Affects Brain Development and Behavior in Mice. International Journal of Molecular Sciences 24, 1–19 (2023). 10.3390/ijms24054688 PMC10002977 36902120

[30] Ho H. A fully automated home cage for long-term continuous phenotyping of mouse cognition and behavior. Cell Reports Methods 3, 100532 (2023). 37533650 10.1016/j.crmeth.2023.100532 PMC10391580

[31] Kazlauckas V. Behavioral and cognitive profile of mice with high and low exploratory phenotypes. Behavioural Brain Research 162, 272–278 (2005). 15970221 10.1016/j.bbr.2005.03.021

[32] Khan R. H., Rhodes J. S., Girard I. A., Schwartz N. E. & Garland T. Does Behavior Evolve First? Correlated Responses to Selection for Voluntary Wheel-Running Behavior in House Mice. Ecological and Evolutionary Physiology 97, 97–117 (2024). 38728689 10.1086/730153

[33] Rezende E. L., Gomes F. R., Chappell M. A. & Garland T. Jr. Running Behavior and Its Energy Cost in Mice Selectively Bred for High Voluntary Locomotor Activity. Physiological and Biochemical Zoology 82, 662–679 (2009). 19799520 10.1086/605917

[34] Goh J. & Ladiges W. Voluntary Wheel Running in Mice. CP Mouse Biology 5, 283–290 (2015). 10.1002/9780470942390.mo140295 PMC4686373 26629772

[35] Meijer J. H. & Robbers Y. Wheel running in the wild. Proc. R. Soc. B. 281, 20140210 (2014). 10.1098/rspb.2014.0210 PMC4046404 24850923

[36] Brooks P. L. & Peever J. A Temporally Controlled Inhibitory Drive Coordinates Twitch Movements during REM Sleep. Current Biology 26, 1177–1182 (2016). 27040781 10.1016/j.cub.2016.03.013

[37] Geisler P., Meier-Ewert K. & Matsubayshi K. Rapid eye movements, muscle twitches and sawtooth waves in the sleep of narcoleptic patients and controls. Electroencephalography and Clinical Neurophysiology 67, 499–507 (1987). 2445541 10.1016/0013-4694(87)90051-4

[38] Sokoloff G. Twitches emerge postnatally during quiet sleep-in human infants and are synchronized with sleep spindles. Current Biology 31, 3426–3432.e4 (2021). 34139191 10.1016/j.cub.2021.05.038 PMC8355086

[39] McShane B. B. Assessing REM Sleep in Mice Using Video Data. Sleep 35, 433–442 (2012). 22379250 10.5665/sleep.1712 PMC3274345

[40] Nestler E. J. & Hyman S. E. Animal models of neuropsychiatric disorders. Nat Neurosci 13, 1161–1169 (2010). 20877280 10.1038/nn.2647 PMC3750731

[41] Bao A.-M. & Swaab D. F. Sex Differences in the Brain, Behavior, and Neuropsychiatric Disorders. Neuroscientist 16, 550–565 (2010). 20889965 10.1177/1073858410377005

[42] Fukui K., Kimura S., Kato Y. & Kohno M. Effects of far infrared light on Alzheimer’s disease-transgenic mice. PLoS ONE 16, e0253320 (2021). 34138944 10.1371/journal.pone.0253320 PMC8211253

[43] Vinberg F. Sensitivity of Mammalian Cone Photoreceptors to Infrared Light. Neuroscience 416, 100–108 (2019). 31400484 10.1016/j.neuroscience.2019.07.047 PMC6815255

[44] Cui X., Niu W. & Yi X. Correlation of behavioural laterality with learning performance of scatter-hoarding rodents. Animal Behaviour 209, 21–27 (2024).

[45] Stieger B., Palme R., Kaiser S., Sachser N. & Richter S. H. When left is right: The effects of paw preference training on behaviour in mice. Behavioural Brain Research 430, 1–11 (2022). 10.1016/j.bbr.2022.113929 35595059

[46] Tang X., Orchard S. M. & Sanford L. D. Home cage activity and behavioral performance in inbred and hybrid mice. Behavioural Brain Research 136, 555–569 (2002). 12429418 10.1016/s0166-4328(02)00228-0

[47] Sotelo M. I. Lateral hypothalamic neuronal ensembles regulate pre-sleep nest-building behavior. Current Biology 32, 806–822 (2022). 35051354 10.1016/j.cub.2021.12.053 PMC10455050

